# Current strategies for monitoring men with localised prostate cancer lack a strong evidence base: observational longitudinal study

**DOI:** 10.1038/sj.bjc.6605181

**Published:** 2009-07-14

**Authors:** C Metcalfe, K Tilling, M Davis, J A Lane, R M Martin, H Kynaston, P Powell, D E Neal, F Hamdy, J L Donovan, on behalf of the ProtecT Study Group

**Affiliations:** 1 Department of Social Medicine, University of Bristol, Canynge Hall, 39 Whatley Road, Bristol, BS8 2PS, UK; 2 Department of Urology, University Hospital of Wales, Heath Park, Cardiff, CF1 4XW, UK; 3 Department of Urology, Freeman Hospital, High Heaton, Newcastle-upon-Tyne, NE7 7DN, UK; 4 Department of Oncology, University of Cambridge, Addenbrooke's Hospital, Hills Road, Cambridge, CB2 0QQ, UK; 5 Nuffield Department of Surgery, Oxford University, John Radcliffe Hospital, Headington, Oxford, OX3 9DU, UK

**Keywords:** disease management, kinetics, PSA, prostatic neoplasms

## Abstract

**Background:**

The UK National Institute for Health and Clinical Excellence (NICE) guidance recommends conservative management of men with ‘low-risk’ localised prostate cancer, monitoring the disease using prostate-specific antigen (PSA) kinetics and re-biopsy. However, there is little evidence of the changes in PSA level that should alert to the need for clinical re-assessment.

**Methods:**

This study compares the alerts resulting from PSA kinetics and a novel longitudinal reference range approach, which incorporates age-related changes, during the monitoring of 408 men with localised prostate cancer. Men were monitored by regular PSA tests over a mean of 2.9 years, recording when a man's PSA doubling time fell below 2 years, PSA velocity exceeded 2 ng ml^–1^ per year, or when his upper 10% reference range was exceeded.

**Results:**

Prostate-specific antigen doubling time and PSA velocity alerted a high proportion of men initially but became unresponsive to changes with successive tests. Calculating doubling time using recent PSA measurements reduced the decline in response. The reference range method maintained responsiveness to changes in PSA level throughout the monitoring.

**Conclusion:**

The increasing unresponsiveness of PSA kinetics is a consequence of the underlying regression model. Novel methods are needed for evaluation in cohorts currently being managed by monitoring. Meanwhile, the NICE guidance should be cautious.

The UK National Institute for Health and Clinical Excellence (NICE) currently recommends that active surveillance is the management strategy of choice for men with localised prostate cancer and a low risk of disease progression; prostate-specific antigen (PSA) <10 ng ml^–1^ and Gleason score of ⩽6 and clinical stage T1-T2a, the so-called ‘low-risk’ prostate cancer (National Institute for Health and Clinical Excellence, 2008). For men with an intermediate risk of disease progression (PSA 10–20 ng ml^–1^ or Gleason score 7 or clinical stage T2b-T2c), NICE recommend that active surveillance should be considered alongside more radical treatment options (National Institute for Health and Clinical Excellence, 2008).

These recommendations are based on expert opinion. They are appealing because most men with localised prostate cancer detected by PSA testing have slow-growing disease, with death due to other causes often intervening before the disease becomes life threatening (Albertsen *et al*, 2005). Although radical treatment is potentially curative for localised disease, there is a significant risk of serious side effects (Frankel *et al*, 2003). Consequently, for men at low risk of disease progression at diagnosis, monitoring strategies (commonly referred to as ‘active surveillance’, ‘active monitoring’ or ‘expectant management’) potentially offer a good balance of risk and benefit, as radical treatment is only undertaken in low-risk men if they subsequently show signs of disease progression (Albertsen *et al*, 2005; Klotz, 2007).

Only the Scandinavian Prostate Cancer Group Study Number 4 (SPCG-4 study) provides relevant trial evidence in this clinical area, and supports a survival advantage for surgery over ‘watchful waiting’ (Bill-Axelson *et al*, 2005). However, watchful waiting does not involve the close monitoring characteristic of modern approaches to conservative management, and the men in SPCG-4 had localised prostate cancer that was clinically detected before PSA testing became common. Consequently these men were older (54% aged 66 years or more) and had a higher Gleason score (33% Gleason score 7 or more) than is typical for men diagnosed after PSA testing (Bill-Axelson *et al*, 2005). Studies of monitoring in PSA-era cohorts are ongoing, but it will be some time before they report clinical outcomes (Donovan *et al*, 2003; Hardie *et al*, 2005; Klotz, 2007; van den Bergh *et al*, 2007). In the meantime, the NICE recommendations are not supported by empirical evidence, but are underpinned by an attempt to apply the SPCG-4 results to a model of a contemporary cohort (Parker *et al*, 2006).

Successful monitoring requires the identification of men whose disease will soon progress, although still allowing for timely, potentially curative treatment. Concurrently, men with indolent disease should not be alerted to the need for clinical reassessment unnecessarily, to avoid invasive investigations and overtreatment (Parker, 2004). The National Institute for Health and Clinical Excellence recommends that men are monitored under the START (Study of Active Surveillance versus Radical Treatement in Patients with Favourable-Risk Prostate Cancer) trial protocol, an ongoing trial where men have their PSA level measured regularly, and undergo prostate biopsy every 2 years (http://www.cancer.gov/clinicaltrials/CAN-NCIC-CTG-PR11). Changes in PSA level, PSA kinetics, are monitored regularly and clinical re-investigation is undertaken if they suggest disease progression. However, this recommendation is also based on expert opinion as two recent systematic reviews showed no universally accepted method of measuring or interpreting PSA level kinetics (Martin *et al*, 2006; van den Bergh *et al*, 2008), and no firm evidence to support any of the wide range of criteria currently being applied in the monitoring programmes.

In this study, two measures of kinetics, PSA doubling time and PSA velocity, and a novel reference range method (Bosch *et al*, 2006) are applied to the PSA measurements taken from a cohort of men who have been managed by monitoring for an average of 2.9 years, allowing us to compare when the different methods raise alerts as monitoring proceeds. Although data on clinical outcomes are not yet available from this or other cohorts of men with screen-detected prostate cancer, this analysis provides important early information on how different approaches to monitoring behave in practice, thus informing the choice of approach for full-scale evaluation.

## Patients and methods

### Patients

The study cohort included 408 men aged 45–70 years diagnosed with histologically proven and clinically localised prostate cancer. Between 2000 and 2008 these men had been invited to undergo a PSA test as part of the UK-based ProtecT (Prostate testing for cancer and Treatment) study (Donovan *et al*, 2003), and then referred for biopsy due to a PSA level of 3.0 ng ml^–1^ or more. This observational study cohort only includes men with clinically localised prostate cancer who refused to be randomly allocated to a treatment in the ProtecT trial, and then chose to be managed by monitoring. The Trent multicentre research ethics committee approved the study, and all participants provided informed consent.

Each man had at least three PSA tests available, including two obtained at diagnosis. At the outset, their mean age was 62 years (s.d. 5.1 years; 90% range 53–69 years) and mean PSA level was 5.60 ng ml^–1^ (s.d. 2.78 ng ml^–1^; 90% range 3.0–10.9 ng ml^–1^). In all, 333 (82%) men had a Gleason score of ⩽6, 73 men a score of 7 and 2 men had a score of 8. The mean duration of monitoring was 2.9 years (s.d. 1.8 years; 90% range 0.7–6.3 years) with PSA being measured an average of 10.0 times (s.d. 5.0 times; 90% range 3–20 times). All men, whether randomly allocated to, or choosing monitoring, are managed according to the same study protocol. In brief, each patient was encouraged to undergo PSA tests every 3 months in year 1, and every 6 months thereafter. Re-biopsy was not routine.

### Methods of measuring PSA change

The PSA doubling-time method uses the rate of increase in PSA measured on a natural logarithmic scale to predict the period of time over which PSA will have doubled, with a short predicted period raising concern. Following the literature, a regression method is used to estimate PSA doubling time. This requires each man to have had his PSA level tested on three or more occasions (Martin *et al*, 2006; van den Bergh *et al*, 2008). A man's log PSA levels are regressed on the dates of measurement: 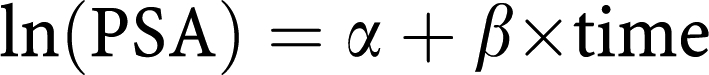 where *α* and *β* are respectively the estimated intercept and gradient of the regression line. A man's PSA doubling time is then calculated as ln(2)/*β*. Each time a new PSA measurement is taken, the doubling time can be recalculated using the new measurement and all measurements collected previously. Concern over the reliability of estimates based on a handful of PSA measures has been expressed, but this has been addressed by considering only those doubling times calculated once the first five measures are available (Ross *et al*, 2004; van den Bergh *et al*, 2007). An alternative approach also requires five measurements to have been taken, but is additionally focused on recent changes in a man's PSA level by using just the most recent five measurements (Ross *et al*, 2004; van den Bergh *et al*, 2007).

In the present implementation of PSA velocity, all the PSA measurements available for a man at a given point are regressed on the measurement times: 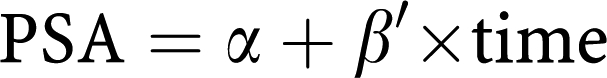 where *α* and *β′* are respectively the estimated intercept and gradient of the regression line. The gradient *β′* is taken as the measure of PSA velocity in ng ml^–1^ per unit time. Again, this method can be reapplied each time a new PSA measurement is taken.

### Longitudinal reference ranges

**Figure 1:**
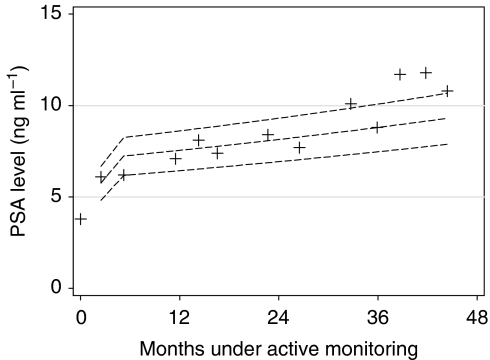
Reference ranges for an individual man, based on age-related changes in a healthy man of the same age and starting PSA level. Plus signs indicate individual measurements of the man's PSA level during monitoring. From the top, the three reference ranges distinguish the PSA level above which the fastest increasing 5, 10 and 20% of similar healthy men will fall due to age-related changes.

Following the research done in other areas of medicine (Tilling, Sterne, and Wolfe, 2001), longitudinal reference ranges have been developed as an alternative to the above methods (Bosch *et al*, 2006; Tilling *et al*, 2009). Parameter values were estimated during a multilevel model analysis of data from the Krimpen (the Netherlands) community-based study of normal age-related changes in PSA levels (Bosch *et al*, 1995). The Krimpen study men were comparable to those in the current cohort, being recruited from all men residing in a defined area, aged 50–75 years at enrolment, and being of predominantly northern European ancestry (Bosch *et al*, 1995). The model parameters are used to produce reference ranges for each man's subsequent period of monitoring, his age and initial PSA level being taken into account (Bosch *et al*, 2006). Figure 1 shows the series of PSA measurements taken for an individual man in the cohort alongside three reference ranges. From the top, these are the reference ranges above which 5, 10 and 20% of men with similar starting PSA levels will fall due to age-related changes in PSA level. Hence, when the man falls above the 10% reference range at 14 months, this suggests that his PSA level has increased at a rate matched or exceeded by only one in ten healthy men. It is postulated that this method may better accommodate the age-related increases in PSA level that occur independently of prostate cancer and thus distinguish deviations from the usual pattern in men with cancer. For this study, longitudinal reference ranges are calculated for each man, based on the man's first two PSA measurements and his age at the start of monitoring. A man is alerted for further investigations when his latest PSA measurement falls above a chosen reference for his current age.

### Statistical methods

A Stata 10 (StataCorp 2007, College Station, TX, USA) batch file was written to apply, retrospectively, each method to each man in the cohort as each new measurement became available, with the occurrence of alerts under the chosen thresholds being recorded. For the purposes of this study, we retrospectively applied the thresholds suggested by Klotz, which gave alerts when the doubling time was calculated to be <2 years and when the PSA velocity was calculated to be >2 ng ml^–1^ per year (Klotz, 2007). Retrospectively applying the reference range method, we considered an alert to have resulted when the man fell into the highest 10% of PSA increases expected from age-related changes in PSA level. This latter threshold is arbitrary, but, for the purposes of this study, it gives an overall alert rate that is comparable to that of the other methods.

## Results

Over the study period, 34% of men (*n*=139) were alerted at least once due to a simple PSA doubling time of <2 years, and 36% of men (*n*=148) were alerted at least once due to a PSA velocity of >2 ng ml^–1^ per year. When doubling time was calculated using just the five most recent measurements, 36% of men (*n*=146) were alerted. The reference range method alerted 34% of men (*n*=140), as on at least one occasion their PSA increase fell into the top 10% of age-related increases for similar men.

**Figure 2:**
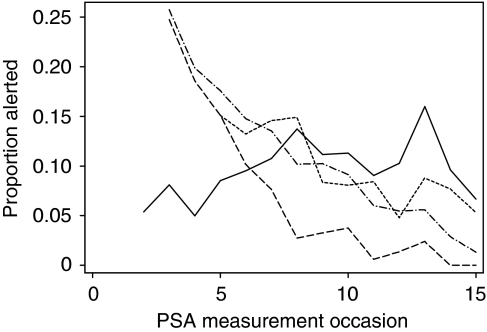
Proportion of men alerted at each measurement occasion by a doubling time, calculated from all data, of <2 years (dashed line), a doubling time, calculated from the five most recent measurements, of <2 years (dotted line), a velocity of more than 2 ng ml^–1^ per year (dot and dash line), and due to exceeding the top 10% reference range (solid line).

Table 1 presents, for each method, the PSA measurements at which alerts occur. The order rather than the timing of measurements is given, and all alerts are included in the table with some men subjected to multiple alerts. The PSA velocity and doubling-time (all measurements) methods both alerted a high proportion of men early on, but a rapidly diminishing proportion as PSA measurements accumulated. Calculating the doubling-time method only when at least five measurements of PSA are available for a man avoids the high initial alert rate but does not avoid the downturn in responsiveness. In contrast, doubling time calculated using the five most recent measures alerts around 15% of men early on, followed by a much shallower decline in responsiveness until around 8% of men are being alerted once a man has accumulated more than ten measurements. No decline in responsiveness is apparent for the reference range method, roughly 8–10% of men being alerted at each measurement. The contrast between the maintained responsiveness of the reference range method and the declining responsiveness of the doubling-time and velocity methods is particularly apparent in Figure 2.

The NICE guidance recommends that men with a doubling time of <3 years should be alerted to the need for clinical review (National Institute for Health and Clinical Excellence, 2008). Applying this criterion to the present cohort leads to about half of men being alerted at least once during the observation period, 49% if doubling time is calculated from all available PSA measurements and 53% if calculated from the five most recent measurements. As PSA measurements accumulate, adoption of the 3-year criterion does not alter the decrease in responsiveness of the doubling time calculated from all available measurements (25% of men alerted after five measurements, 9% after ten measurements and 3% after fifteen measurements), and the same largely maintained responsiveness is seen with doubling time calculated from the most recent five measurements (21% of men alerted after ten measurements and 20% after fifteen measurements).

**Figure 3:**
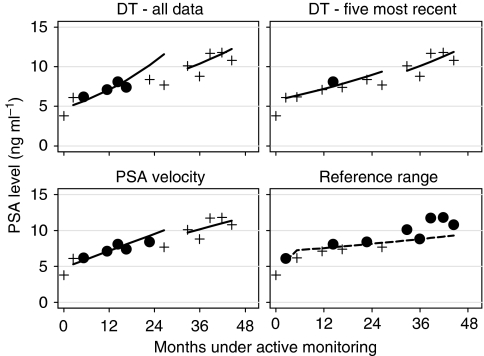
Prostate-specific antigen levels over time for an example case managed by monitoring. Solid circles indicate measurements at which the applied criterion caused an alert, plus symbols indicating the other measurements. Solid lines indicate the regression lines fitted to calculate doubling time (DT) and velocity at the sixth and eleventh measurements. The dotted line is the longitudinal reference range threshold above which 10% of similar men are predicted to fall at each measurement time.

Figure 3 again presents the series of PSA measurements from our exemplar man, this time with all the investigated methods applied. The two regression lines used in calculating doubling time and the two regression lines used in calculating velocity at the sixth and eleventh measurements are shown. Measurements at which a method applied with the chosen threshold causes an alert are shown as dots, other measurements are shown as plus signs. The doubling-time method applied to all measurements and PSA velocity alert the man repeatedly early on, but not at all after 2 years of monitoring. Allowing the doubling-time method to cause an alert only once five measurement are available would have alerted the man at his fifth and sixth measurements only, although the doubling-time method applied to just the most recent five measurements would have alerted the man at his fifth measurement only. The man also exceeds his 10% upper reference range at his third and fifth measurements and, in contrast to the doubling-time and velocity methods, he continues to exceed it during his third and fourth years of monitoring.

## Discussion

We have compared measures of PSA kinetics to a novel longitudinal reference range approach, examining when these different methods alert men whose localised prostate cancer is being monitored. Prostate-specific antigen doubling time and PSA velocity alert a high proportion of men initially but quickly become unlikely to alert men as the PSA measurements accumulate. In contrast, the reference range method appeared equally sensitive to changes in PSA level as monitoring progressed.

Prostate-specific antigen doubling time and PSA velocity are calculated by regressing a man's series of PSA levels on the dates of measurement. Early on, the calculations will be unreliable as they will be overly susceptible to the well-known short-term variation in PSA level. That unreliability is likely to be behind the initial high rate of alerts with the regression-based methods. Once a man has accumulated a number of measurements, regression will provide a better indication of the overall trend in his PSA level, but will become increasingly insensitive to any upturn in PSA level. This problem has been described in a study of PSA velocity measured using pre-operative PSA measurements (Yu *et al*, 2006). We observed that the problem of increasing insensitivity was partly avoided by using recent PSA measurements in the calculation of doubling time (Yu *et al*, 2006), although this may allow too much influence to short-term variations in the PSA level. Fundamentally, the best-fit regression line for a series of PSA measurements will be influenced by the balance of measurements taken during the periods of steady PSA level and any subsequent upturn. Consequently regression-based methods will be slow to highlight changes in PSA level when used in long-term surveillance programmes, with this insensitivity being resistant to minor modifications to the method.

Prostate-specific antigen doubling time and PSA velocity alerted around 35% of men at least once during an average of 2.9 years of monitoring, comparable to the 39% of men alerted by a doubling time of <2 years and the 49% of men alerted by a velocity of more than 2 ng ml^–1^ per year recently reported for the Sunnybrook cohort after an average of 7 years monitoring (Klotz, 2007). The Sunnybrook cohort includes men in their 70s and 80s, and with a median age of 70, that cohort is older than the one examined in this study. The two cohorts are comparable in having around 80% of men with Gleason tumor grade of ⩽6. With a much longer average period of monitoring, a greater proportion of men might have been expected to have been alerted in the Sunnybrook cohort, but this does not seem to be the case for doubling time in particular (Klotz, 2007). Either the same men are being alerted repeatedly as monitoring proceeds, or doubling time is showing the same insensitivity in the Sunnybrook cohort, alerting very few men as measures accumulate.

Prostate-specific antigen doubling time and PSA velocity both require the use of statistical software each time a new measurement of PSA becomes available, and this may be difficult to achieve in outpatient clinics (Schröder, de Vries, and Bangma, 2003). Although there are calculation tools for those methods on the internet (Martin *et al*, 2006; van den Bergh *et al*, 2008), the reference range approach is even more convenient in requiring computer software only at the point when the first two measurements of PSA are available. The longitudinal reference ranges can then be plotted for an individual patient, and subsequent measures of PSA compared with them. This method of monitoring PSA levels over time seemed to remain sensitive to changes for the whole period observed in this study. Furthermore, plotting a reference range over time, as in Figure 1, shows the increase in PSA level expected with advancing age in a healthy man. This may provide reassurance, as what might be a worrying trend upwards is shown to be consistent with the expected age-related increases for a healthy man of similar age and starting PSA level (Latini *et al*, 2007; Pickles *et al*, 2007).

The short follow-up and the lack of clinical progression data are the limitations of this study, as clinically relevant thresholds cannot be determined and the sensitivity and specificity cannot be compared between methods. There are currently no published data available with both longitudinal PSA measurements and progression in untreated men with screen-detected localised prostate cancer. The required data are accumulating in a number of studies around the world, notably one North American (Klotz, 2007) and three European studies (Donovan *et al*, 2003; Hardie *et al*, 2005; van den Bergh *et al*, 2007), but it will be a number of years yet before these studies are able to report clinical outcome information. Even then, it may be found that many cohort members have switched to radical treatment without firm evidence of progressing disease, as occurred in previous studies of monitoring in men with clinically detected disease (Martin *et al*, 2006). In this context, this study represents valuable preliminary work, indicating important problems with currently promoted PSA-based criteria, which need to be addressed now for men whose prostate cancer is currently being managed by monitoring (Donovan *et al*, 2003; Hardie *et al*, 2005; Klotz, 2007; van den Bergh *et al*, 2007).

Selection bias is a further potential limitation, as a declining alert rate in men monitored for long periods may be expected if men alerted early are then treated radically and thus removed from our cohort. In other words, those men still being managed by monitoring after seven or eight PSA measurements may be a selected group with very stable PSA levels. This is unlikely to be a full explanation of our findings, as the reference range method is detecting a similar proportion of men with increasing PSA levels over the whole observation period.

In conclusion, there is little research evidence to guide the use of PSA kinetics for monitoring men with screen-detected and localised prostate cancer, and this study highlights problems with the methods currently recommended by NICE. Prostate-specific antigen doubling time and PSA velocity seem to rapidly become insensitive to changes as a man undergoes repeated tests, and this may mean they are unsuitable for the long-term monitoring/surveillance of men with localised prostate cancer. There is now a need for novel methods of monitoring, such as the reference range method evaluated here, which should be evaluated in ongoing studies of monitoring for validation against clinical progression events and disease outcome. The NICE guidance needs to make men and clinicians, using methods of surveillance/monitoring for prostate cancer, aware of the uncertainties about PSA kinetics and of the caution required in interpreting PSA changes.

## Figures and Tables

**Table 1:** Number of alerts occurring with PSA velocity, doubling time applied to all data (DT all), doubling time applied to the most recent five measures (DT 5), and the reference range method as additional tests become available

**PSA test**	**PSA velocity >2 ng ml^–1^ per year (%)**	**DT all <24 months (%)**	**DT 5 <24 months (%)**	**Reference range in top 10% (%)**	**Number tested**
1	—	—	—	—	408
2	—	—	—	22 (5)	408
3	105 (26)	101 (25)	—	33 (8)	408
4	76 (20)	71 (19)	—	19 (5)	382
5	64 (18)	55 (15)	55 (15)	31 (9)	364
6	48 (15)	33 (10)	43 (13)	31 (10)	325
7	39 (14)	22 (8)	42 (15)	31 (11)	288
8	26 (10)	7 (3)	38 (15)	35 (14)	255
9	22 (10)	7 (3)	18 (8)	24 (11)	215
10	17 (9)	7 (4)	15 (8)	21 (11)	186
11	10 (6)	1 (1)	14 (8)	15 (9)	166
12	8 (5)	2 (1)	7 (5)	15 (10)	146
13	7 (6)	3 (2)	11 (9)	20 (16)	125
14	3 (3)	0	8 (8)	10 (10)	104
15	1 (1)	0	4 (5)	5 (7)	75
16+	10	0	21	26	239

Men may be alerted more than once. Dashes indicate the early PSA tests at which a method cannot indicate an alert.
